# The occurrence of Adrenocorticotropic hormone-independent Cushing's syndrome in a woman with the history of papillary thyroid carcinoma: a case report

**DOI:** 10.1186/s13256-021-02684-x

**Published:** 2021-03-11

**Authors:** Mashallah Tabatabaizadeh, Sara Hasibi Taheri, Mohammad Eydi, Mohammad Shayestehpour

**Affiliations:** 1 Autoimmune Disease Research Center, Kashan University of Medical Sciences, Kashan, IR Iran; 2 Department of Internal Medicine, Kashan University of Medical Sciences, Kashan, IR Iran; 3 Department of General Surgery, Kashan University of Medical Sciences, Kashan, IR Iran

**Keywords:** Cushing's syndrome, Adrenal adenoma, Papillary thyroid carcinoma (PTC)

## Abstract

**Background:**

Thyroid papillary carcinoma is one of the most common endocrine tumors, and it accounts for 85% of thyroid tumors. Adrenocorticotropic hormone (ACTH)-independent Cushing's syndrome is a rare disease. In this case report, we discuss a very rare case of coexistence of papillary thyroid carcinoma and Cushing's syndrome with an adrenal origin.

**Case presentation:**

The patient was a 33-year-old Iranian/Persian woman with a history of papillary thyroid carcinoma treated with iodine 131 three years ago. She presented with weight gain, amenorrhea, and mood disorders in the last six months. She was diagnosed with an ACTH-independent Cushing's syndrome due to benign adrenal adenoma and underwent laparoscopic adrenalectomy surgery. The symptoms of the syndrome were disappeared after the surgery.

**Conclusions:**

ACTH-independent Cushing's syndrome due to adrenal tumor and papillary thyroid cancer occurs sporadically. The co-occurrence of two endocrine tumors with different origins is rare. It is recommended that the occurrence of other endocrine neoplasms be considered when an endocrine tumor is diagnosed.

## Introduction

Endocrine tumors make up about 4% of the body's tumors. Thyroid papillary carcinoma is one of the most common endocrine tumors, and it accounts for 85% of thyroid tumors. Cushing's syndrome is a rare disease with a prevalence of 1–2 cases per 100,000 people in the population. Only 10% of patients have Adrenocorticotropic hormone (ACTH)-independent Cushing's syndrome, and a majority of those are women. Exogenous Cushing is the most common cause of Cushing Syndrome. Adrenocortical adenoma, adrenocortical carcinoma, and adrenal micronodular hyperplasia can cause ACTH-independent Cushing's syndrome [1].

Cushing's syndrome is caused by chronic exposure of the body to glucocorticoids; therefore, the increase of cortisol level affects several physiological systems. The majority of clinical signs and symptoms observed in Cushing's syndrome are non-specific and include manifestations such as obesity, diabetes, hypertension, hirsutism, and depression. Cushing's diagnosis should be considered when multiple manifestations are found in the same patient, especially specific manifestations, including the fragility of the skin, accompanied by easy bruising, purple wide striae, and symptoms of proximal myopathy [1].

There are few case reports on the coexistence of thyroid papillary carcinoma and adrenal adenoma. In a case study reported in Israel, a 72-year-old man had a rare coexistence of papillary, medullary, follicular thyroid carcinoma, and Cushing's syndrome caused by the left adrenal adenoma [2]. In another case report in Turkey, a 40-year-old patient had both a rare adrenal mass and papillary thyroid carcinoma [3]. Tung et al. were reported the co-occurrence of bilateral papillary thyroid cancer (PTC) cancer with adrenal adenoma leading to Cushing's syndrome in a 41-year-old man diagnosed with Carney syndrome [4]. Carney complex is a hereditary multiple endocrine neoplasia syndrome characterized by spotty skin pigmentation and mucosal surfaces, cardiac and noncardiac myxomatous tumors, and multiple endocrine tumors. In this case report, we discuss a very rare case of coexistence of papillary thyroid carcinoma and Cushing's syndrome with an adrenal origin.

## Case presentation

The patient was a 33-year-old Iranian/Persian woman with a history of bilateral multifocal papillary thyroid carcinoma and left-sided cervical lymphadenopathy. She underwent a total thyroidectomy and treated with iodine 131 at a dose of 150 ml 3 years ago. Subsequently, she gradually was developed weakness, lethargy, facial swelling (moon face), and abdominal obesity. The patient's weight was increased from 70 to 113 kg over 3 years, and nutrition counseling did not control her weight gain. The patient was referred to Shahid Beheshti Hospital in Kashan, Iran. She was completed a consent form. The patient did not have a family history of thyroid disease or a malignancy. She was living in Kashan and was not an addict or a smoker. The patient was taking Levothyroxine (100 μg daily), Sertraline (100 mg bidaily), Propranolol (20 mg bidaily), and Respridone (1 mg bidaily). The patient did not have a history of steroid use. The patient experienced irregular menstrual cycles with intervals of 90 days and the duration of each cycle equal to 4 days. She had low-volume bleeding, and over time, the intervals between her menstrual cycles were increased. Eventually, the menstrual cycle was interrupted, and the patient was developed secondary amenorrhea. She was experienced mood swings, depression, and aggression during this period that did not improve despite treatment. Her blood pressure was 150/50, and her Body mass index (BMI) was 42. Hirsutism was observed on her face, breast, and around the navel. Purple striae on the abdomen, dryness all over the skin, multiple ecchymoses on the leg (easy bruising), a and muscle strength of 4/5 on both lower limbs were evident.

Firstly, laboratory tests, including Thyroid-stimulating Hormone (TSH), Follicle-stimulating Hormone (FSH), Luteinizing Hormone (LH), Prolactin, Estradiol, urine-free cortisol (24 hours), and overnight dexamethasone were requested for the patient (Table 1). The screening tests of Cushing's syndrome were positive; therefore, the low-dose dexamethasone test was performed to confirm the diagnosis. The patient was treated for two days with 0.5 mg dexamethasone every 6 hours, and the cortisol level (8 AM) was above 50 nmol/L [4].

**Table 1:** The result of laboratory tests in a woman with the history of papillary thyroid carcinoma and ACTH-independent Cushing's syndrome

Reference range	Result	Test
0.4–4.0 mIU/L	0.2 mIU/L	TSH
<33 ng/ml	0.04 ng/ml	Tg
1.2–12.5 IU/L	0.5 IU/L	FSH
3.7–7.8 IU/L	0.1 IU/L	LH
30–400 pg/ml	<5 pg/ml	Estradiol
< 14 ng/mL	16.77 ng/ml	Prolactin
800–2000 ml/day	1590 ml/day	Urine volume (24hrs)
800–1800 mg/24 hours	610 mg/24 hours	Urine creatinine (24hrs)
< 20 μg/24 hour	446 μg/24 hours	Urine Free cortisol (24hrs)
< 2 μg/dL	12.42 μg/dL342.792nmol/L	Overnight Dexamethasone test Cortisol (8 AM)
<5 μg/dL	16.78 μg/dL = 463.128 nmol/L	Low dose Dexamethasone test Cortisol (8 AM)
10–60 pg/ml	1.2 pg/ml	ACTH
70–100 mg/dL	143 mg/dL	FBS
<150 mg/dL	256 mg/dL	Triglyceride
<200 mg/dL	295 mg/dL	Cholesterol
85–125 mg/dL	125 mg/dL	LDL
40–80 mg/dL	37 mg/dL	HDL
136–145 mmol/L	138 mmol/L	Na
3.5–5 mmol/L	4.07 mmol/L	K

TSH: Thyroid-stimulating Hormone, Tg: Thyroglobulin, FSH: Follicle-stimulating Hormone, LH: Luteinizing Hormone, ACTH: Adrenocorticotropic hormone, FBS: Fasting Blood Sugar, LDL: Low-Density Lipoprotein, HDL: High-Density Lipoprotein, Na: sodium, K: potassium

**Fig. 1:**
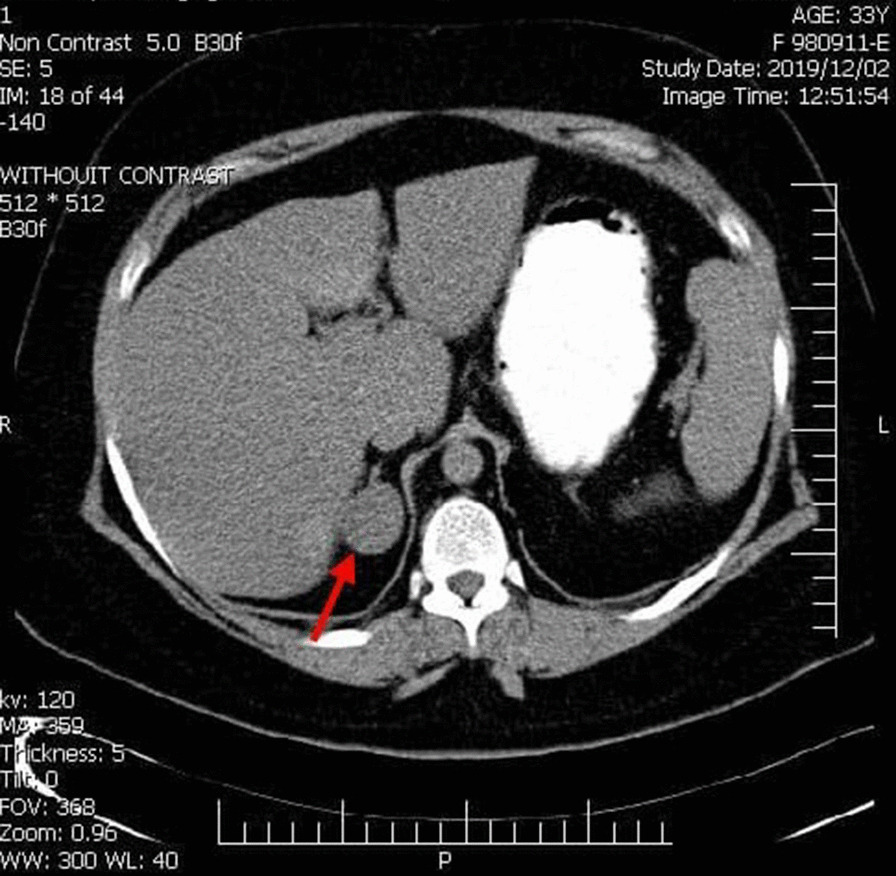
Spiral computed tomography of the abdomen using injection contrast: arrow shows a 3.5 cm diameter mass with a regular margin and significant hemogenic density in the right adrenal gland

The cortisol level was 463 nmol/L; therefore, Cushing's syndrome was proposed for the patient. The ACTH level was measured to differentiate Cushing's syndrome from Cushing's disease. It was below 5 pg/ml, and the disease was considered as ACTH-independent Cushing's syndrome. An adrenal mass was observed in a computerized tomography (CT) scan of the adrenal gland (Fig 1). The patient underwent laparoscopic right adrenalectomy and was removed a mass with 3.5 cm in diameter and weighing 18 g. Pathologic evaluation was suggested the benign adrenal adenoma. The mass was a fatty tissue without necrosis and hemorrhage. During 6 days after surgery, the patient was treated with an injection of 100 mg daily hydrocortisone. She was treated with oral prednisolone (5 mg daily) after discharge.

After 4 months, most of the symptoms of Cushing's syndrome, including weight gain, menstrual irregularities, hirsutism, and abdominal striae were partially cured. The patient's blood pressure, electrolytes, and TSH levels were normal, while she was receiving prednisolone and levothyroxine 100 micrograms per day. No mass was observed on a CT scan of the patient's abdomen after three months; therefore, the surgery was successful.

## Discussion

Cushing's syndrome indicates chronic exposure of the body to glucocorticoids for any reason. The disorder can be ACTH-dependent (pituitary adenoma, non-pituitary ACTH-secreting tumor) and ACTH-independent (adrenal adenoma or carcinoma, adrenal hyperplasia).

Cushing's syndrome is generally one of the rare diseases, and its prevalence is 1–2 cases per 100,000 population. Only 10% of patients have ACTH-independent Cushing's, and a majority of those are women [1].

The coexistence of PTC bilateral multi-focal cancer and ACTH-independent Cushing's syndrome with the origin of unilateral adrenal adenoma has not been reported in the literature. In a study conducted in Imam Khomeini Hospital, Tehran, Iran, none of the 253 cases with Cushing's syndrome had a history of PTC [5].

In the present case report, we found a 33-year-old woman with a history of papillary thyroid cancer and symptoms of Cushing's, abdominal obesity, and amenorrhea, who had an adrenal adenoma. Two weeks after laparoscopic adrenalectomy surgery, the level of 24-hour urinary cortisol significantly was decreased to the normal level. After 4 months, most of the symptoms of Cushing's syndrome were partially cured. The result of the bone scan was normal; therefore, it was ensured that recurrence or metastasis of papillary carcinoma did not occur. The co-occurrence of two endocrine tumors was not related to each other and it is a rare event.

## Conclusion

ACTH-independent Cushing's syndrome due to adrenal tumor and papillary thyroid cancer occur sporadically. The co-occurrence of two endocrine tumors with different origins is rare. It is recommended that the occurrence of other endocrine neoplasms be considered when an endocrine tumor is diagnosed.

## Data Availability

No additional file is available for this study; all the data are included in the manuscript.

## Abbreviations

ACTH: Adrenocorticotropic hormone
PTC: Papillary thyroid carcinoma
